# Bone Morphogenetic Protein 7 Gene Delivery Improves Cardiac Structure and Function in a Murine Model of Diabetic Cardiomyopathy

**DOI:** 10.3389/fphar.2021.719290

**Published:** 2021-10-08

**Authors:** Mitchel Tate, Nimna Perera, Darnel Prakoso, Andrew M. Willis, Minh Deo, Osezua Oseghale, Hongwei Qian, Daniel G Donner, Helen Kiriazis, Miles J. De Blasio, Paul Gregorevic, Rebecca H. Ritchie

**Affiliations:** ^1^ Drug Discovery Biology, Monash Institute of Pharmaceutical Sciences, Parkville, VIC, Australia; ^2^ Heart Failure Pharmacology, Baker Heart and Diabetes Institute, Melbourne, VIC, Australia; ^3^ School of Biosciences, The University of Melbourne, Parkville, VIC, Australia; ^4^ Centre for Muscle Research, Department of Anatomy and Physiology, The University of Melbourne, Parkville, VIC, Australia; ^5^ Preclinical Microsurgery and Imaging, Baker Heart and Diabetes Institute, Melbourne, VIC, Australia; ^6^ Department of Cardiometabolic Health, The University of Melbourne, Parkville, VIC, Australia; ^7^ Department of Pharmacology, Monash University, Clayton, VIC, Australia; ^8^ Baker Heart and Diabetes Institute, Melbourne, VIC, Australia; ^9^ Biochemistry and Molecular Biology, Monash University, Clayton, VIC, Australia; ^10^ Department of Neurology, The University of Washington, Seattle, WA, United States

**Keywords:** bone morphogenetic protein 7, diabetic cardiomyopathy, adeno-associated virus, cardiac remodelling, gene therapy

## Abstract

Diabetes is a major contributor to the increasing burden of heart failure prevalence globally, at least in part due to a disease process termed diabetic cardiomyopathy. Diabetic cardiomyopathy is characterised by cardiac structural changes that are caused by chronic exposure to the diabetic milieu. These structural changes are a major cause of left ventricular (LV) wall stiffness and the development of LV dysfunction. In the current study, we investigated the therapeutic potential of a cardiac-targeted bone morphogenetic protein 7 (BMP7) gene therapy, administered once diastolic dysfunction was present, mimicking the timeframe in which clinical management of the cardiomyopathy would likely be desired. Following 18 weeks of untreated diabetes, mice were administered with a single tail-vein injection of recombinant adeno-associated viral vector (AAV), containing the BMP7 gene, or null vector. Our data demonstrated, after 8 weeks of treatment, that rAAV6-BMP7 treatment exerted beneficial effects on LV functional and structural changes. Importantly, diabetes-induced LV dysfunction was significantly attenuated by a single administration of rAAV6-BMP7. This was associated with a reduction in cardiac fibrosis, cardiomyocyte hypertrophy and cardiomyocyte apoptosis. In conclusion, BMP7 gene therapy limited pathological remodelling in the diabetic heart, conferring an improvement in cardiac function. These findings provide insight for the potential development of treatment strategies urgently needed to delay or reverse LV pathological remodelling in the diabetic heart.

## Introduction

The global prevalence of diabetes is estimated to increase to 700 million by 2045, significantly impacting global health expenditure (Saeedi et al., 2019). Diabetes substantially increases the risk of developing diabetic complications, including cardiovascular diseases (D’souza et al., 2011). One consequence of the global epidemic of diabetes has been the increased appreciation of a distinct form of heart failure in these patients, termed diabetic cardiomyopathy, which is independent of other cardiovascular comorbidities. In fact, epidemiological studies suggested that diabetic patients are at 2-3 fold increased risk of developing heart failure than non-diabetics (Kannel et al., 1974; Regan et al., 1977; D’elia et al., 1979), and this holds true over 4 decades later (Marwick et al., 2018).

The underlying pathophysiological mechanisms responsible for the development and progression of diabetic cardiomyopathy remain poorly understood, although it is widely appreciated that chronic exposure to the diabetic milieu leads to degenerative changes in the heart that have limited capacity for repair (Tate et al., 2017; Ritchie and Abel, 2020). The diabetic heart is particularly susceptible to extracellular matrix remodelling, triggered initially by changes in cardiomyocyte biology (Huynh et al., 2014; Tate et al., 2017; Ritchie and Abel, 2020). Apoptosis, a form of programmed cell death, is important in the transition from compensated hypertrophy to eventual heart failure in the diabetic heart (Bugger and Abel, 2014; Ritchie and Abel, 2020). Recent reports suggest apoptosis is causal in the progression of diabetic cardiomyopathy, and the progressive loss of cardiomyocytes and contractile units leads to increased collagen deposition and irreversible replacement fibrosis (Huynh et al., 2012; Huynh et al., 2013).

Bone morphogenetic proteins (BMP) belong to the transforming growth factor-beta (TGF-β) super family and provide an array of often paradoxical cellular actions, dependent on location and context of stimuli (Massagué, 2012). The human genome encodes >20 BMP ligands that confer important effects on cellular behaviour, including cellular proliferation and differentiation, highlighted by high levels of evolutionary conservation (Rahman et al., 2015). Whereas TGF-β is a major biological signal that initiates a pro-fibrotic response, BMP7 appears to counterbalance these actions, and may in fact be antifibrogenic, restoring vascular stability and limiting capillary rarefaction in diabetic nephropathy (Wang et al., 2001; Pofi et al., 2017). Furthermore, BMP7 activity is strictly regulated by several secreted antagonists, including Gremlin, noggin and follistatin (Iemura et al., 1998; Fürthauer et al., 1999; Merino et al., 1999). In fact, levels of these endogenous BMP7 inhibitors are increased in diabetes (Wang et al., 2001), with tubule-specific knockdown of Gremlin conferring improvements in acute renal fibrosis (Church et al., 2017). As expected, given its influence on multiple physiological processes, BMP7 has been implicated in the pathogenesis of several diseases (Wang et al., 2001; Boon et al., 2011; Merino et al., 2016; Pofi et al., 2017).

To date, few studies have addressed the role of BMPs in cardiovascular diseases, and to our knowledge, this is the first study to assess the therapeutic potential of targeting BMP7 signalling in diabetic cardiomyopathy. In patients with aortic stenosis, and mice subjected to transverse aortic constriction, pressure overload resulted in decreased levels of BMP7 and pSmad1/5/8 (the intracellular effectors of BMP7) and an increase in TGF-β and pSmad2/3 (Merino et al., 2016). Moreover, BMP7 expression is negatively correlated with cardiac fibrosis and diastolic dysfunction (Merino et al., 2016). Furthermore, in cardiomyocytes treated with recombinant BMP2 or BMP7, treatment limited apoptosis and cardiomyocyte hypertrophy, both *via* the Smad1/5/8 mediated pathway (Izumi et al., 2001; Merino et al., 2016). In a pre-diabetic murine model of diabetes, BMP7 treatment elicited a beneficial impact on specific aspects of cardiac remodelling, namely cardiomyocyte apoptosis and cardiac fibrosis, which conferred improvements in cardiac function (Urbina and Singla, 2014).

Given the lack of studies exploring the potential protective actions of BMP7 in diabetes and/or cardiac pathologies, we sought to investigate whether manipulation of BMP7 signalling would confer an improvement on adverse cardiac remodelling in the diabetic heart. In order to negate the complex and context-dependent nature of TGF-β/BMP7 pathway, we implemented a cardiac-directed adeno-associated viral (AAV) vector-mediated gene delivery therapeutic approach into our study design.

## Methods

### Animals

All activities involving the use of animals for research were approved by the Alfred Medical Research Education Precinct (AMREP) Animal Ethics Committee and were conducted according to guidelines of the National Health and Medical Research Council (NH&MRC) of Australia for animal experimentation. FVB/N mice were sourced from the AMREP Animal Services. Mice had free access to food and water and were housed at 22 ± 1°C on a 12-h light/dark cycle.

### Experimental Design

**FIGURE 1:**
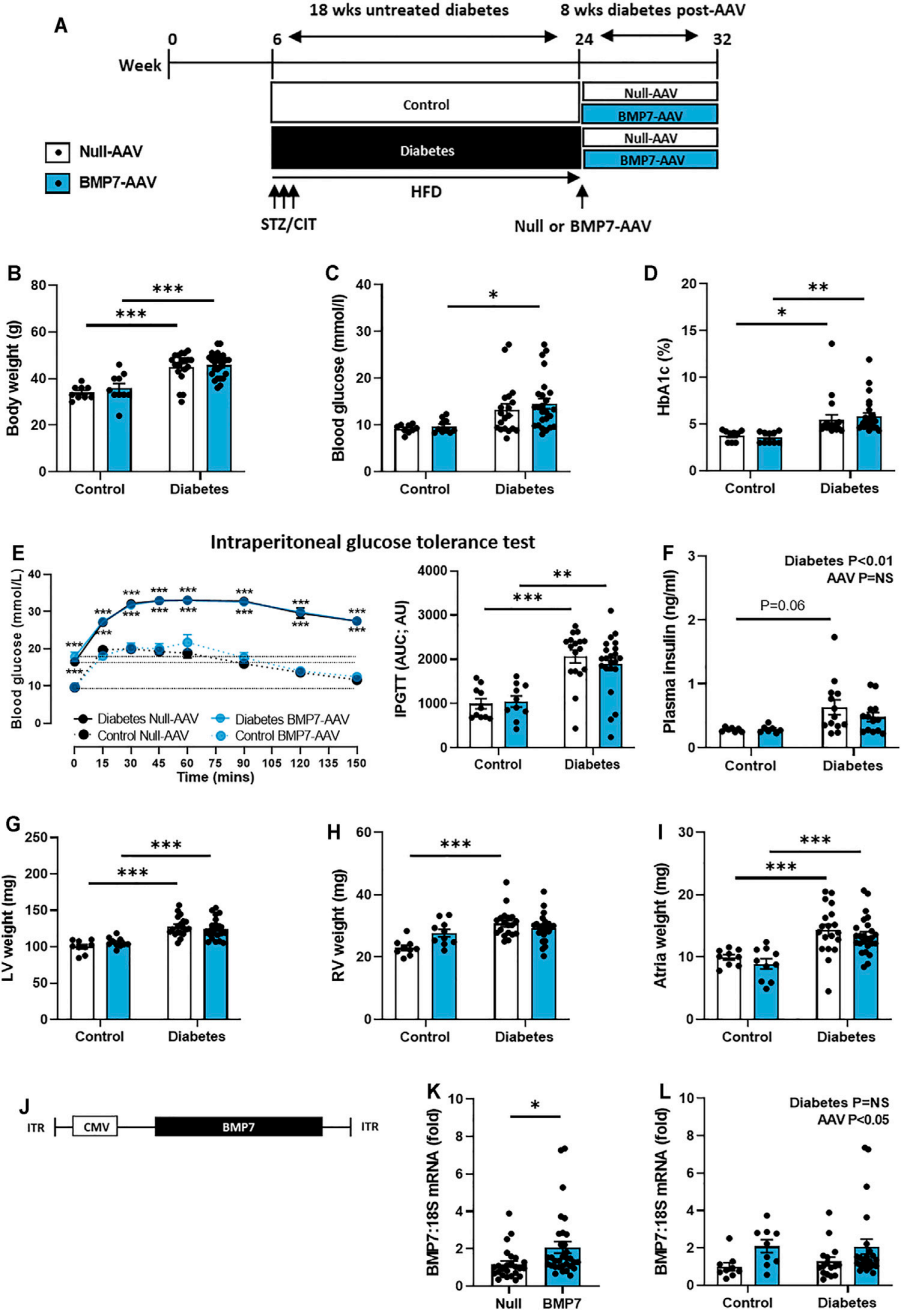
The effect of rAAV6-BMP7 gene therapy on diabetes-induced systemic and morphological changes. **(A)** Overview of experimental protocol, **(B)** body weight, **(C)** blood glucose, **(D)** glycated haemoglobin (HbA1c), **(E)** intraperitoneal glucose tolerance test (IPGTT; time course and AUC)**, (F)** plasma insulin, **(G)** LV weight, **(H)** RV weight, **(I)** atria weight. **(J)** Schematic representation of the AAV vector used to achieve cardiac-selective overexpression of BMP7, containing the CMV promotor, the BMP7 gene and inverted terminal repeats (ITRs). **(K)** Cardiac mRNA expression of BMP7 in rAAV6-Null and rAAV6-BMP7 groups (data pooled for Control and Diabetes) and **(L)** in four treatment groups. Data are presented as mean ± SEM. *n* = 7–25 per group (see individual data points). *p < 0.05, **p < 0.01, ***p < 0.001. **(B–D, F–I, L)** Two-way ANOVA followed by Tukey’s *post hoc* test, **(E)** repeated measures two-way ANOVA followed by Tukey’s post *hoc* test, or **(K)** unpaired *t*-test. AAV, adeno-associated virus; AUC, area-under-the-curve; BMP7, bone morphogenetic protein 7; CIT, sodium citrate; CMV, cytomegalovirus virus; LV, left ventricle; RV, right ventricle.

For all experiments we have included flow charts for the reporting of animal numbers and analysis in preclinical studies (Supplementary Figure S1). The main aim of this study was to investigate the effect of cardiac-selective rAAV6-BMP7 gene therapy on cardiac structure and function in an experimental model of diabetic cardiomyopathy. Accordingly, our primary endpoint was impact of rAAV6-BMP7 gene therapy on markers of left ventricular (LV) diastolic function (E/A ratio, e’/a’ ratio, isovolumic relaxation time (IVRT) and deceleration time). Male, 6-week-old FVB/N mice received three consecutive-daily i. p. injections of streptozotocin (STZ; 55 mg/kg body weight, in 0.1 mol/L citric acid vehicle, pH4.5; Sigma) combined with 18 weeks of high-fat diet (42% energy intake from lipids, SF04-001, Specialty Feeds) to induce diabetes (Figure 1A). Control mice received sham vehicle injections and normal chow-diet. Diabetes was confirmed by measuring blood glucose every 2 weeks *via* the saphenous vein using a glucometer (Accu-Chek, Roche). At 18 weeks of diabetes, mice received a one-off injection of rAAV6-BMP7 or rAAV6-Null (Figure 1A), as described below.

### Adeno-Associated Virus Generation and Administration

The BMP7 construct, previously utilised in skeletal muscle (Winbanks et al., 2013), was cloned into an rAAV6 expression plasmid consisting of a cytomegalovirus (CMV) promoter/enhancer packaged into pseudotype-6 capsids (rAAV6), as previously described (Gregorevic et al., 2004; Winbanks et al., 2013; Prakoso et al., 2017; Prakoso et al., 2020; Prakoso et al., 2021a). The purified vector preparations were titered with a customised sequence-specific quantitative PCR-based reaction. rAAV6-BMP7 or transgene-null vector (rAAV6-Null) were administered *via* a single tail-vein injection at a dose of 2 × 10^11^ vector genomes (in 150 μL of saline; Figure 1A). This dose of AAV6 vector with CMV promoter has previously been demonstrated to achieve robust expression in cardiac muscle only, as for other constructs including PI3Kp110α, OGA and OGT (Weeks et al., 2012; Prakoso et al., 2017; Prakoso et al., 2020; Prakoso et al., 2021a).

### Intraperitoneal Glucose Tolerance Test

Intraperitoneal glucose tolerance tests (IPGTT) were conducted 1 week prior to endpoint. Prior to IPGTT, mice were fasted for 5 h and had their baseline blood glucose level recorded. At time 0, a 25% glucose solution (4 μL/g, Baxter, Viaflex®) was injected *via* a single i.p bolus, after which blood glucose measurements were obtained *via* tail vein bleeds at 15, 30, 45, 60, 90 and 120 min. Area-under-the-curve (AUC) was calculated to determine the rate of glucose clearance.

### Tissue Collection

At study end, animals received an endpoint dose of ketamine/xylazine (85/8.5 mg/kg i.p.). Blood samples were collected by cardiac puncture into heparinised tubes and centrifuged at 3,000 g for 15 min to obtain separate cell and plasma fractions. Plasma samples were collected for subsequent analysis. The heart and other organs including gastrocnemius, tibia, kidney and liver, were collected. The heart was dissected into the atria, right ventricle and left ventricle. A section of the LV was collected for histology and a section was snap-frozen in liquid nitrogen and stored at −80°C for RNA extraction and RTPCR. Glycated haemoglobin (HbA1c) was measured at study end using the Cobas b 101 POC system (Roche). Circulating plasma insulin concentrations at endpoint were measured by mouse-specific insulin ELISA kit (cat no. 80-INSMSU-E01, ALPCO), as per manufacturer’s instructions.

### Echocardiography

To obtain measures of LV function, echocardiography was performed in anaesthetised mice (ketamine/xylazine/atropine: 80/8/0.96 mg/kg i.p.) at study endpoint utilising a Philips iE33 ultrasound (North Ryde, NSW, Australia) with 15 MHz linear (M-mode) and 12 MHz sector (Doppler) transducer, as previously described (Prakoso et al., 2017; Tate et al., 2019; De Blasio et al., 2020; Prakoso et al., 2020; Prakoso et al., 2021a). LV chamber dimensions and fractional shortening were assessed from M-mode imaging. LV filling was assessed using transmitral Doppler; the ratio of early (E) and late (A) peak blood flow velocities (E/A ratio), deceleration time and IVRT were measured (Prakoso et al., 2017; Tate et al., 2019; De Blasio et al., 2020; Prakoso et al., 2020; Prakoso et al., 2021a).

### Histology and Immunohistochemistry

Following excision, hearts were weighed and fixed in 10% neutral-buffered formalin solution (NBF, Australian Biostain, Melbourne, VIC, Australia). All histological analyses were performed using paraffin-embedded LV sections (4 µm). Cardiomyocyte cross-sectional area was determined by haematoxylin and eosin (H&E; Sigma-Aldrich, St Louis, MO, United States) staining, analysing cells with centrally located nuclei measuring the smallest cross-sectional diameter of each cardiomyocyte. Individual areas and widths of 60–100 cardiomyocytes per animal were measured. The picrosirius red stain (0.1% w/v; Sigma-Aldrich, St Louis, MO, United States) was conducted to assess LV interstitial fibrosis (20X magnification) and LV perivascular fibrosis (40X) magnification. Ten random fields of view were imaged per LV for both brightfield and polarised light to quantify total collagen and collagen type I and collagen type III fibres, respectively. Cardiomyocyte apoptosis was assessed by TUNEL staining (CardioTACs, Trevigen, MD, United States). Ten random fields of view were imaged per LV (20X magnification) for apoptosis with TUNEL positive cells quantified as a percentage of total cardiac cell number per field of view. All images were imaged using a BX43 microscope (Olympus, Japan) and analysed blinded from the experimental group using ImageJ, as previously described (Prakoso et al., 2017; Tate et al., 2019; De Blasio et al., 2020; Prakoso et al., 2020; Prakoso et al., 2021a).

### RNA Isolation and Quantitative RT-PCR

LV and gastrocnemius tissue were collected, snap-frozen in liquid nitrogen, and stored at −80°C for gene expression analysis (Prakoso et al., 2017; Tate et al., 2019; De Blasio et al., 2020; Prakoso et al., 2020; Prakoso et al., 2021a). RNA was extracted from frozen LV homogenate using TRIzol® reagent (Invitrogen Life Technologies, Mount Waverley, VIC, Australia), and cDNA synthesised by reverse transcription (Invitrogen Life Technologies, Mount Waverley, VIC, Australia), both techniques carried out in accordance with manufacturer’s instructions. mRNA expression of BMP7 (F: GGC​TGG​CAG​GAC​TGG​ATC​AT; R: GGC​GCA​CAG​CAG​GGC​TTG​G) was analysed in LV tissue and gastrocnemius muscle by real-time reverse transcription-polymerase chain reaction (RT-PCR) using fluorescent SYBR® Green (Applied Biosystems, Scoresby, Victoria, Australia). *18S* (F: TGT​TCA​CCA​TGA​GGC​TGA​GAT​C; R: TGG​TTG​CCT​GGG​AAA​ATC​C), whose expression was shown to remain unaltered between experimental groups, was used for normalisation. Primers were generated from murine sequences published on GenBank. Quantitative analysis was performed using QuantStudio 7 Flex Real-Time PCR System and software (ThermoFisher Scientific), using the 2^−ΔΔCt^ method to detect fold differences relative to the control group.

### Statistical Analysis

Data were analysed with GraphPad Prism 9.0.0 statistical software package (GraphPad, La Jolla, CA, United States). All data are presented as mean ± SEM. Differences between groups were compared using an unpaired t-test or two-way analysis of variance (ANOVA) with Tukey’s pos*t hoc* test. Statistical significance was considered at *p <* 0.05.

## Results

### Systemic Characteristics Following rAAV6-BMP7 Treatment

As depicted in Figure 1A, diabetes was induced in 6-week old male mice using a combination of low-dose STZ (3 consecutive-daily injections) and high-fat diet. Diabetes was left untreated for 18 weeks to replicate the clinical scenario, where patients often have evidence of cardiac structural and functional changes before any intervention can be made. Mice then received a single tail-vein injection of rAAV6-BMP7 or rAAV6-Null vector. At end point, following 8 weeks of rAAV6-BMP7 treatment, body weight, blood glucose and HbA1c levels were all significantly increased in diabetic mice (Figures 1B–D). rAAV6-BMP7 treatment had no impact on these parameters (Figures 1B–D). Another measure of animal size, tibial length, was the same in all groups (Table 1). Diabetic mice exhibited impaired glucose intolerance at study end, as demonstrated by increased area-under-the-curve (AUC; Figure 1E). In addition, there was also a significant elevation in plasma insulin levels in diabetic mice compared with control mice (Figure 1F). Again, these measures were unchanged in rAAV6-BMP7-treated animals.

**TABLE 1:** Organ weights and systolic heart function in control and diabetic mice following rAAV6-BMP7 or rAAV6-Null treatment.

	Control		Diabetes	
	rAAV6-Null	rAAV6-BMP7	rAAV6-Null	rAAV6-BMP7
Organ weights				
*n*	9	10	19	25
Tibial length (mm)	17.9 ± 0.2	17.9 ± 0.1	18.0 ± 0.1	18.1 ± 0.1
Kidney weight (left; mg)	215 ± 3	239 ± 8	250 ± 10	251 ± 6
Lungs weight (mg)	157 ± 6	160 ± 4	199 ± 8^***^	186 ± 4^*^
Liver weight (g)	1.56 ± 0.04	1.68 ± 0.06	2.62 ± 0.12^***^	2.56 ± 0.10^***^
Gastrocnemius weight (mg)	190 ± 11	180 ± 4	199 ± 4	195 ± 6
Spleen weight (mg)	97 ± 2	106 ± 4	154 ± 5^***^	142 ± 4^***^
Pancreas weight (mg)	250 ± 11	278 ± 13	248 ± 11	268 ± 16
Fat pad weights				
Pericardial fat weight (mg)	14.9 ± 2.4	26.5 ± 6.9	40.8 ± 4.8^**^	34.7 ± 3.9
Perirenal fat weight (g)	0.68 ± 0.11	0.82 ± 0.12	1.49 ± 0.14^**^	1.60 ± 0.11^**^
Inguinal fat weight (g)	0.79 ± 0.15	0.89 ± 0.16	2.56 ± 0.22^***^	2.55 ± 0.21^***^
M-mode echocardiography				
*n*	9	10	17	22
Heart rate (bpm)	310 ± 15	345 ± 20	361 ± 13	372 ± 14
AWd (mm)	0.70 ± 0.01	0.70 ± 0.01	0.77 ± 0.02	0.77 ± 0.01^*^
PWd (mm)	0.70 ± 0.01	0.71 ± 0.02	0.77 ± 0.02	0.78 ± 0.01
LVEDD (mm)	4.11 ± 0.05	4.14 ± 0.04	4.22 ± 0.04	4.21 ± 0.05
LVESD (mm)	2.85 ± 0.08	2.84 ± 0.09	2.87 ± 0.05	2.89 ± 0.07
Fractional shortening (%)	31 ± 2	31 ± 2	32 ± 1	31 ± 1

Data are presented as mean ± SEM. Two-way ANOVA followed by Tukey’s post *hoc* test.

*p < 0.05, **p < 0.01, ***p < 0.001 vs. corresponding Control. AWd, anterior wall thickness during diastole; LVEDD, LV end diastolic dimension; PWd, posterior wall thickness during diastole; LVESD, LV end systolic dimension.

Several organs and fat pads were larger in diabetic mice compared to control mice, including the lungs, liver, spleen, pancreas, pericardial fat, perirenal and inguinal fat (Table 1). None of these measures were impacted by rAAV6-BMP7 treatment (Table 1). In the heart, diabetic animals had increased LV, RV and atrial weights, although this was not changed in mice treated with rAAV6-BMP7 (Figures 1G–I).

The plasmid packaged into the rAAV6 vector consisted of the CMV-promoter and the BMP7 gene construct (or Null construct), as illustrated in Figure 1J. In cardiac tissue at study end, there were significantly higher levels of BMP7 mRNA expression in mice that received rAAV6-BMP7, compared to those that received rAAV6-Null (Figure 1K, pooling control and diabetic mice within each of the two AAVs used). Plotting the data in Figure 1K as the four distinct experimental groups, there were no differences in BMP7 mRNA levels between diabetic and control mice (Figure 1L). Due to tropism of the AAV6 serotype to striated muscle, mRNA expression was examined in the gastrocnemius muscle of mice in this study to determine if the AAV intervention was cardiac selective or also increased BMP7 expression in muscle. At study end, there was no significant increase in BMP7 mRNA expression in the mice that received rAAV6-BMP7, compared to those that received rAAV6-Null (Supplementary Figure S2).

### Cardiac Function Following rAAV6-BMP7 Treatment

**FIGURE 2:**
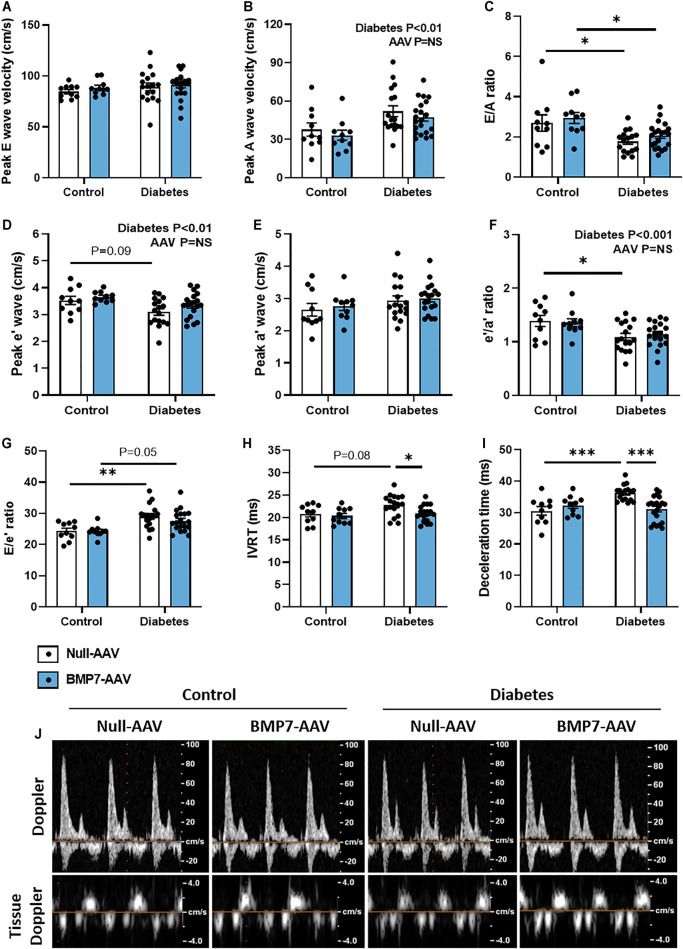
The effect of rAAV6-BMP7 gene therapy on diabetes-induced LV diastolic dysfunction. Doppler echocardiography was used to derive **(A)** peak E wave velocity, **(B)** peak A wave velocity, **(C)** E/A ratio, **(D)** peak e’ wave velocity, **(E)** peak a’ wave velocity, **(F)** e’/a’ ratio, **(G)** E/e’ ratio, **(H)** isovolumic relaxation time (IVRT), **(I)** deceleration time, as depicted in the **(J)** representative images. Data are presented as mean ± SEM. *n* = 10–21 per group (see individual data points). *p < 0.05, **p < 0.01, ***p < 0.001. Two-way ANOVA followed by Tukey’s post *hoc* test. AAV, adeno-associated virus; BMP7, bone morphogenetic protein 7.

At study end, there was clear evidence of diastolic dysfunction in diabetic mice, as assessed by Doppler and tissue Doppler echocardiography (Figure 2). There was no change in peak E wave velocity with diabetes (Figure 2A), however there was an increase in peak A wave velocity in diabetic mice, compared to control mice (Figure 2B), that led to a significant reduction in E/A ratio (Figure 2C). This was also corroborated by a reduction in peak e’ in diabetic mice (Figure 2D) that resulted in a reduction in e’/a’ ratio in diabetic mice (Figure 2F; no change in Peak a’ between groups, Figure 1E). There was an increase in the E/e’ ratio with diabetes (Figure 2G). Moreover, there was a prolongation in both isovolumic relaxation time (IVRT; Figure 2H) and deceleration time (Figure 2I) in diabetic mice. Importantly, these measures were attenuated in mice that received rAAV6-BMP7 treatment (Figures 2H,I). Representative Doppler and tissue Doppler images for each group are shown (Figure 2J). LV diastolic dysfunction is typically followed by systolic dysfunction as diabetic cardiomyopathy progresses. At the time point studied in this model there was no evidence of systolic dysfunction, as highlighted by no change in fractional shortening between groups (Table 1). However, several of the LV wall and chamber dimensions assessed (anterior wall dimension, LV end-diastolic dimension and posterior wall dimension) were larger in diabetic mice compared to control mice (Table 1), logical since the hearts from diabetic mice were larger than those from control mice (Figures 1G–I). Importantly, heart rate remained the same between groups (Table 1).

### Characterisation of Cardiac Structural Changes Following rAAV6-BMP7 Treatment

**FIGURE 3:**
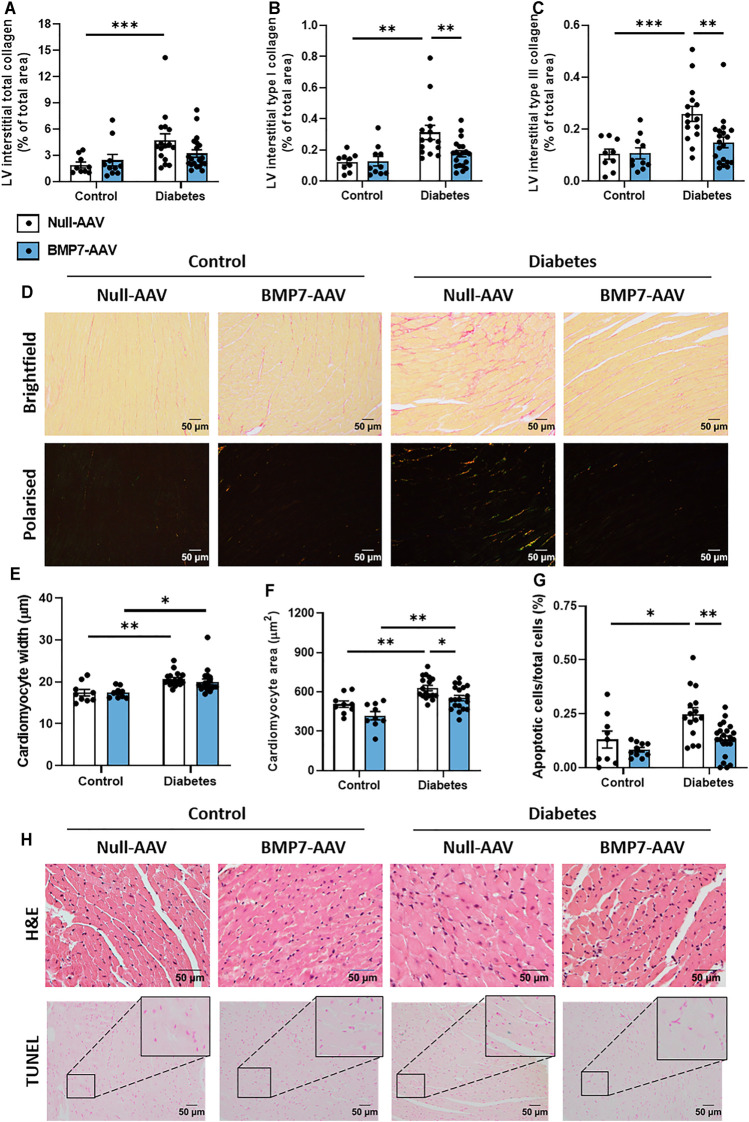
The effect of rAAV6-BMP7 gene therapy on diabetes-induced structural changes. Quantification of picrosirius red-stained LV cross sections. **(A)** Total interstitial collagen content using brightfield imaging, interstitial type I **(B)** and type III **(C)** collagen content using polarised light. **(D)** Representative images of picrosirius red-stained collagen in LV cross sections (top panel, brightfield; bottom panel, polarised light; scale bar: 50 µm). Quantification in LV cross sections of **(E)** cardiomyocyte width and **(F)** cardiomyocyte area in haematoxylin and eosin-stained samples, and **(G)** TUNEL-positive apoptotic cells. **(H)** Representative images of haematoxylin and eosin-stained LV sections (top panel) and TUNEL-positive apoptotic cells (bottom panel; scale bar: 50 µm). Data are presented as mean ± SEM. *n* = 9–20 per group (see individual data points). *p < 0.05, **p < 0.01, ***p < 0.001. Two-way ANOVA followed by Tukey’s *post hoc* test. AAV, adeno-associated virus; BMP7, bone morphogenetic protein 7; H&E, haematoxylin & eosin; LV, left ventricle.

Total LV interstitial collagen content was increased at study endpoint in diabetic mice, when compared to control mice (Figure 3A). Polarised light was then used to specifically quantify type I and type III collagen in the picrosirius red-stained sections. Interestingly, there was a significant elevation in both type I and type III interstitial collagen with diabetes, and these levels were attenuated in diabetic mice treated with rAAV6-BMP7 (Figures 3B,C). Representative brightfield and polarised light images for each group are shown (Figure 3D). Total LV perivascular collagen content was increased at study endpoint in diabetic mice when compared to control mice (Supplementary Figure S3A). Perivascular type I and type III collagen content was increased in diabetic mice when compared to controls (Supplementary Figures S3B–C), however there was no effect of rAAV6-BMP7 administration on the content of perivascular fibrosis. Another common structural hallmark of diabetic cardiomyopathy is cardiomyocyte hypertrophy. Diabetic mice had a significant increase in cardiomyocyte width and area (Figures 3E,F). Importantly, treatment with rAAV6-BMP7 in diabetic mice led to a reduction in cardiomyocyte area (Figure 3F). Diabetes-induced cardiomyocyte apoptosis was evident by an increase in LV TUNEL-positive-stained cells, which was ameliorated following treatment with rAAV6-BMP7 gene therapy (Figure 3G). Representative H&E and TUNEL-stained images for each group are shown (Figure 3H).

## Discussion

The main finding of this study is that a cardiac-selective gene therapeutic approach, designed to overexpress BMP7, offers cardioprotection in a murine model of diabetic cardiomyopathy. rAAV6-BMP7 treatment had no impact on the underlying diabetes phenotype, but treatment did improve several detrimental structural changes characteristic of disease pathology. The link between diabetes and an increased propensity to develop heart failure is well established (Kannel et al., 1974), and highlights the need to identify new treatment strategies that are designed specifically to resolve the underlying pathology responsible for driving disease progression (Marwick et al., 2018; Ritchie and Abel, 2020).

Gene therapy is a very promising approach to treat heart failure, as it offers the possibility to correct abnormalities in the structure and function of the heart. Several studies have assessed the therapeutic potential of gene transfer in experimental animal models (Prakoso et al., 2017; Tate et al., 2017; Prakoso et al., 2021a), as well as in heart failure patients (Jessup et al., 2011; Hammond et al., 2016), with AAVs shown to possess desirable properties including serotypes that have high degrees of tropism for the heart (Greenberg, 2017; Prakoso et al., 2021b). Despite these advances, little data exists in the diabetic heart, and this is the first study to use gene therapy to target BMP7 signalling in this setting. TGF-β and BMP7 belong to the same superfamily of proteins but exert opposing actions by signalling through distinct Smad proteins (Weiskirchen and Meurer, 2013). TGF-β is the primary driver of adverse cardiac remodelling in both rodents and humans, whilst BMP-7 is a known anti-fibrotic cytokine (Wang et al., 2006). Given the wide array of physiological actions TGF-β and BMP signalling exerts, one major advantage of implementing an organ-specific approach is to limit the detrimental on-target actions in other organs.

In the present study, BMP7 mRNA expression was elevated in the hearts of mice that received rAAV6-BMP7, compared to mice that received rAAV6-Null (Figure 1). Given that this AAV6 serotype has tropism to striated muscle we investigated BMP7 mRNA expression in the gastrocnemius muscle from mice in this study. These analyses were undertaken to determine whether transgene expression *via* this intervention was limited to the myocardium or also inclusive of skeletal muscle. There was no significant increase in BMP7 gene expression in the gastrocnemius muscle between any of the treatment groups and no difference when we pooled the control and diabetic mice within each of the two AAV (BMP7 and null) groups (Supplementary Figure S2). Due to the unavailability of an effective antibody for BMP7 or downstream signalling proteins, detection of successful transduction of the BMP7 at the protein level has not been possible. This is a significant limitation of the current study and other studies that report BMP7 protein abundance. That said, our lab has previously engineered several gene therapies using the same plasmid backbone and virus subtype (i.e., the only difference being the gene of interest), and has proven successful at achieving overexpression of the gene of interest only in a cardiac-selective manner (Prakoso et al., 2017; Prakoso et al., 2020; Prakoso et al., 2021a). Furthermore, in these previous studies, mRNA levels of the gene of interest have correlated with protein levels as these could be measured using a suitable antibody (Prakoso et al., 2017; Prakoso et al., 2020; Prakoso et al., 2021a). Although we have not been able to confirm cardiac-selective expression of BMP7 at the protein level in this study, the beneficial effects observed following rAAV6-BMP7 treatment are likely due to successful transduction of the BMP7 gene, and therefore, these results remain of significant interest.

Heart failure incidence is much higher in patients with diabetes, with a 1% increase in HbA1c conferring an 8% increased risk of developing heart failure, independent of other factors (Stratton et al., 2000; Ritchie and Abel, 2020). Recent clinical trials of glucose-lowering therapies including glucagon-like peptide 1 receptor agonists (LEADER trial) and sodium–glucose cotransporter-2 inhibitors (EMPA-REG OUTCOME trial), report beneficial cardiovascular disease outcomes in type 2 diabetes patients (Zinman et al., 2015; Marso et al., 2016). Despite this, scope remains for new treatment strategies that specifically target the pathological features of diabetic cardiomyopathy once they have manifested (particularly in patients who do not tolerate these new glucose-lowering approaches (Prakoso et al., 2021b)). In this study, rAAV6-BMP7 treatment had no impact on glycaemic control, with blood glucose and HbA1c levels unaltered following treatment (Figure 1).

Typical disease progression includes a lengthy asymptomatic/subclinical period, characterised by the presence of cardiac fibrosis, ventricular stiffness and associated diastolic dysfunction, which is not typically detected with the usual battery of clinical tests (Jia et al., 2018). Heart failure with preserved ejection fraction (HFpEF) typically advances to heart failure with reduced ejection fraction following the development of systolic dysfunction (Tate et al., 2017). In this study, systolic dysfunction was not observed at the time point studied (Table 1). At study end, following 26 weeks of diabetes, diastolic dysfunction was present in this model, as highlighted by changes in E/A ratio, E’/A′ ratio, E/e’ ratio, deceleration time and IVRT, assessed by Doppler and tissue Doppler echocardiography (Figure 2). This is consistent with previous reports (Tate et al., 2019). In mice that received rAAV6-BMP7, treatment was able to significantly improve several of the markers of diastolic function (Figure 2). In another study, treatment with recombinant BMP7 protein in a rodent model of prediabetes, was able to confer improvements in diastolic function (Urbina and Singla, 2014); in that study recombinant BMP7 protein administration commenced at the same time as induction of diabetes. Importantly, this study provides the first evidence that BMP7 treatment, given after functional changes have manifest, therefore mimicking the clinical scenario, may have a beneficial impact on the diabetic heart.

Several drivers exist in the diabetic heart that promote cardiac fibrosis and cardiomyocyte hypertrophy, key characteristics of disease pathology. TGF-β signalling plays a pivotal role in the pro-hypertrophic response. In models of pressure overload, canonical TGF-β-mediated signalling led to a maladaptive pro-hypertrophic response (Koitabashi et al., 2011). In the present study, cardiomyocytes were enlarged in diabetic hearts, likely *via* TGF-β-mediated mechanism, an effect attenuated in rAAV6-BMP7-treated mice (Figure 3). In transgenic mice overexpressing TGF-β, interstitial fibrosis was also reported alongside cardiomyocyte hypertrophy (Rosenkranz et al., 2002). In the present study, diabetes induced increases in LV interstitial and perivascular collagen content were consistent with other diabetic mouse models (Huynh et al., 2013; De Blasio et al., 2015; Prakoso et al., 2017; Tate et al., 2019; De Blasio et al., 2020; Prakoso et al., 2020; Prakoso et al., 2021a). Importantly, both type I and type III LV interstitial collagen levels were reduced in rAAV6-BMP7-treated mice (Figure 3), however we did not see amelioration of perivascular fibrosis deposition with BMP7 gene therapy (Supplementary Figure S3). The visualisation of collagen fibres with picrosirius red relies on the birefringence of collagen fibres and is one of the most understood histochemical methods to specifically determine collagen fibres in tissue samples. The determination of collagen fibre type using picrosirius red, combined and enhanced with polarised light, is an inexpensive, simple, and currently a very reliable method to perform quantitative detection of well-defined type I collagen, and green-stained type III collagen, in paraffin-embedded sections (Cury et al., 2013; Cury et al., 2016; Rittié, 2017; Liu et al., 2021). In addition, this technique is currently widely used within the literature to measure individual collagen fibre types within histological samples. TGF-β stimulates the activation and proliferation of cardiac fibroblasts thereby increasing the pool of pro-fibrotic cardiac myofibroblasts (Zeisberg et al., 2003; Koitabashi et al., 2011). Several studies have documented the key role of BMP7 to antagonise TGF-β-induced myofibroblast transition (Zeisberg et al., 2003). Importantly, in the kidney, overexpression of BMP7 in transgenic mice protects against the development of diabetic nephropathy (Wang et al., 2006). Moreover, recombinant BMP7 therapy inhibits fibrosis in several rodent models of renal fibrosis, including the STZ diabetic mouse (Sugimoto et al., 2007). More recently, BMP7 has been reported to counteract TGF-β-induced accumulation of myofibroblasts and ECM production in the heart (Zeisberg et al., 2003; Weiskirchen and Meurer, 2013).

Cardiac apoptosis can trigger collagen deposition to maintain cardiac function, however maladaptive remodelling, a consequence of long-term exposure to the diabetic milieu, is a major cause of LV wall stiffening and the development of diastolic dysfunction. Cardiomyocyte apoptosis has previously been reported in both experimental models of diabetes and in patients with diabetes (Frustaci et al., 2000; Fiordaliso et al., 2006). In the present study, diabetic hearts exhibited an increased number of TUNEL-positive apoptotic cardiac cells, an effect reduced significantly following rAAV6-BMP7 administration (Figure 3). Our laboratory has previously reported that cardiac overexpression of constitutively active phosphoinositide 3-kinase (PI3-K), in the diabetic heart, protects against cardiomyocyte apoptosis (Prakoso et al., 2017; Prakoso et al., 2020). Furthermore, BMP7 has been shown to modulate PTEN (a tumour suppressor protein and endogenous inhibitor of PI3-K) and the Akt pathway (Urbina and Singla, 2014; Chattopadhyay et al., 2017), whilst activation of the Smad1 pathway by BMP2 inhibited serum deprivation-induced apoptosis in neonatal cardiomyocytes, *via* the induction of anti-apoptotic protein Bcl2 (Izumi et al., 2001). The cell target of our BMP7 AAV gene delivery is cardiomyocytes, as the AAV6 serotype shows tropism for striated muscle (as opposed to other cell types in the heart such as fibroblasts). This is also evident from our data, in which both cardiomyocyte apoptosis is assessed (CardioTACS is a TUNEL-based assay optimised for this purpose) and haematoxylin and eosin histological analysis of cardiomyocyte dimensions. BMP7-treated pre-diabetic mice showed significantly increased levels of anti-inflammatory IL-10 and reductions in TNFα-induced cardiomyocyte apoptosis (Urbina and Singla, 2014). In addition, the results from our picrosirius red staining indicates that BMP7 administration may have an effect on cardiac fibroblasts. However, more studies are required to elucidate the exact mechanism (and precise cellular targets) of BMP7’s protective actions following production by cardiomyocytes.

Our study provides evidence that rAAV6-BMP7 treatment confers cardioprotection in an experimental model of diabetic cardiomyopathy, although further work is required to elucidate the underlying mechanisms. The increase in cardiac fibrosis is a significant issue in the diabetic cardiomyopathy milieu. Whilst current strategies such as angiotensin-converting enzyme inhibitors (ACEi) and angiotensin II receptor blockers (ARBs) are attractive approaches for slowing the progression of diabetic cardiomyopathy, cardiac fibrosis can continue to accumulate as these drugs do not reverse the diabetes-induced changes in the heart. We have shown in this study that BMP7 AAV gene therapy has some efficacy in attenuating diabetes-induced changes in cardiac structure and function and provides the pre-clinical groundwork into the potential use of cardiac-targeted gene therapy for limiting the progression of diabetic cardiomyopathy, such as we have recently postulated (Prakoso et al., 2021b). Whilst our treatment may not exceed ACEi or ARBs, potential future strategies may use BMP7 either as a stand-alone therapy or in combination with other medications to provide a greater cardiac benefit.

## Data Availability

The original contributions presented in the study are included in the article/Supplementary Files, further inquiries can be directed to the corresponding author.

## Supplementary Material

Supplementary Figure S1 Flow chart of animal use and analysis based on the CONsolidated Standards of Animal Experiment ReporTing (CONSAERT) template for preclinical studies.

Supplementary Figure S2
**(A)** Gastrocnemius muscle mRNA expression of BMP7 in Control and Diabetes groups (data pooled) and **(B)** in the four treatment groups. Data are presented as mean ± SEM. *n* = 7–25 per group (see individual data points). **(A)** Unpaired t-test, **(B)** two-way ANOVA followed by Tukey’s post hoc test, AAV, adeno-associated virus; BMP7, bone morphogenetic protein 7.

Supplementary Figure S3 Quantification of picrosirius red-stained LV cross sections. **(A)** Total perivascular collagen content using brightfield imaging, perivascular type I **(B)** and perivascular type III **(C)** collagen content using polarised light. **(D)** Representative images of picrosirius red-stained perivascular LV cross sections (top panel, brightfield; bottom panel, polarised light; scale bar: 50 µm). Data are presented as mean ± SEM. *n* = 8–21 per group (see individual data points). **(A–C)** Two-way ANOVA followed by Tukey’s post hoc test, AAV, adeno-associated virus; BMP7, bone morphogenetic protein 7.

## Abbreviations

AAV, adeno-associated virus; ANOVA, analysis of variance; AUC, area-under-the-curve; AWd, anterior wall thickness during diastole; BMP, bone morphogenetic protein; CMV, cytomegalovirus; H&E, haematoxylin and eosin; HbA1c, glycated haemoglobin; IPGTT, intraperitoneal glucose tolerance test; IVRT, isovolumic relaxation time; ITRs, inverted terminal repeats; LV, left ventricle/ventricular; LVEDD, LV end diastolic dimension; LVESD, LV end systolic dimension; PWd, posterior wall thickness during diastole; SEM, standard error of the mean; STZ, streptozotocin; TGF-β, transforming growth factor-beta; PI3-K, phosphoinositide 3-kinase.
