# Supplementary figures and images for: Bone Morphogenetic Protein 7 Gene Delivery Improves Cardiac Structure and Function in a Murine Model of Diabetic Cardiomyopathy
Source: Front Pharmacol. 2021 Oct 8;12:719290. doi: 10.3389/fphar.2021.719290 (PMC8532155; doi:10.3389/fphar.2021.719290)

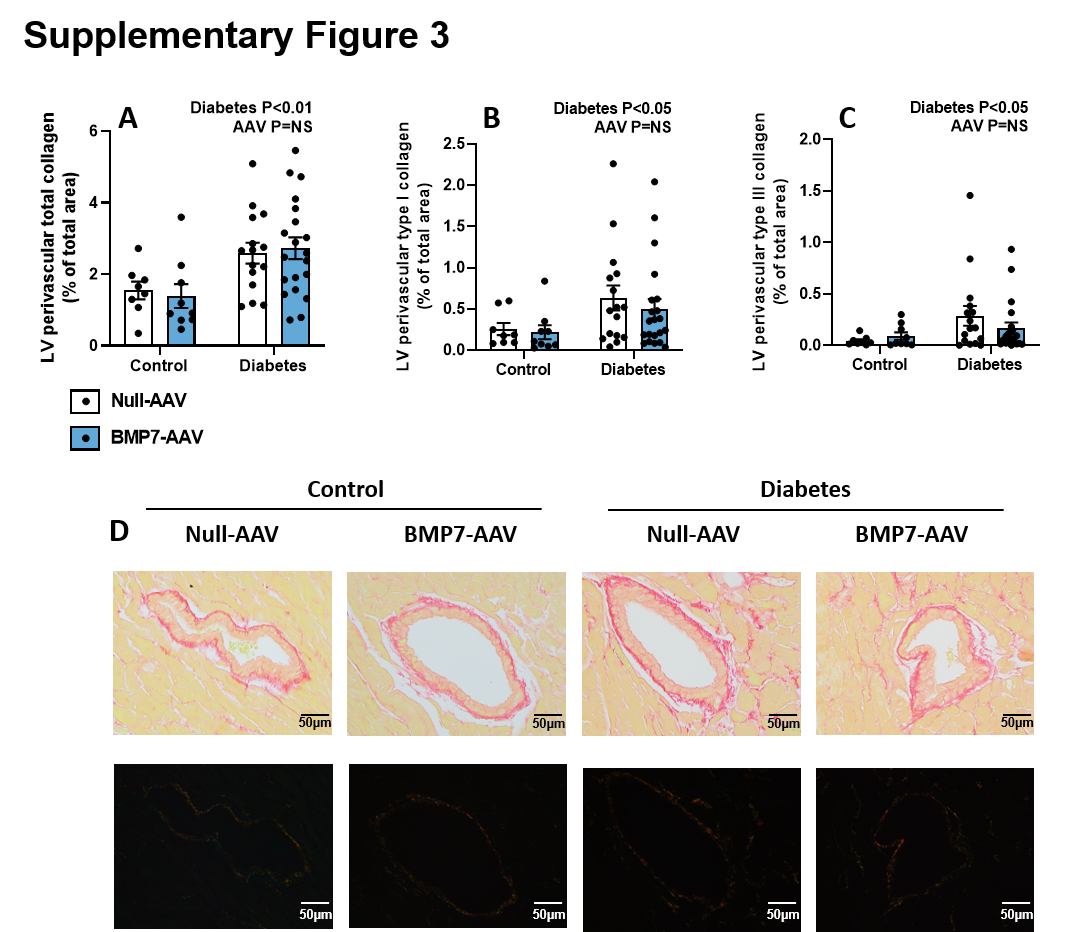

Supplement: Supplementary file 1 [file Image3.TIF]

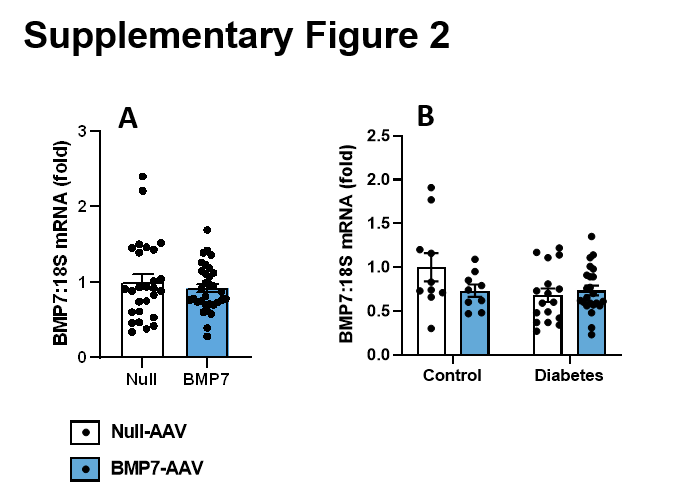

Supplement: Supplementary file 2 [file Image2.TIF]

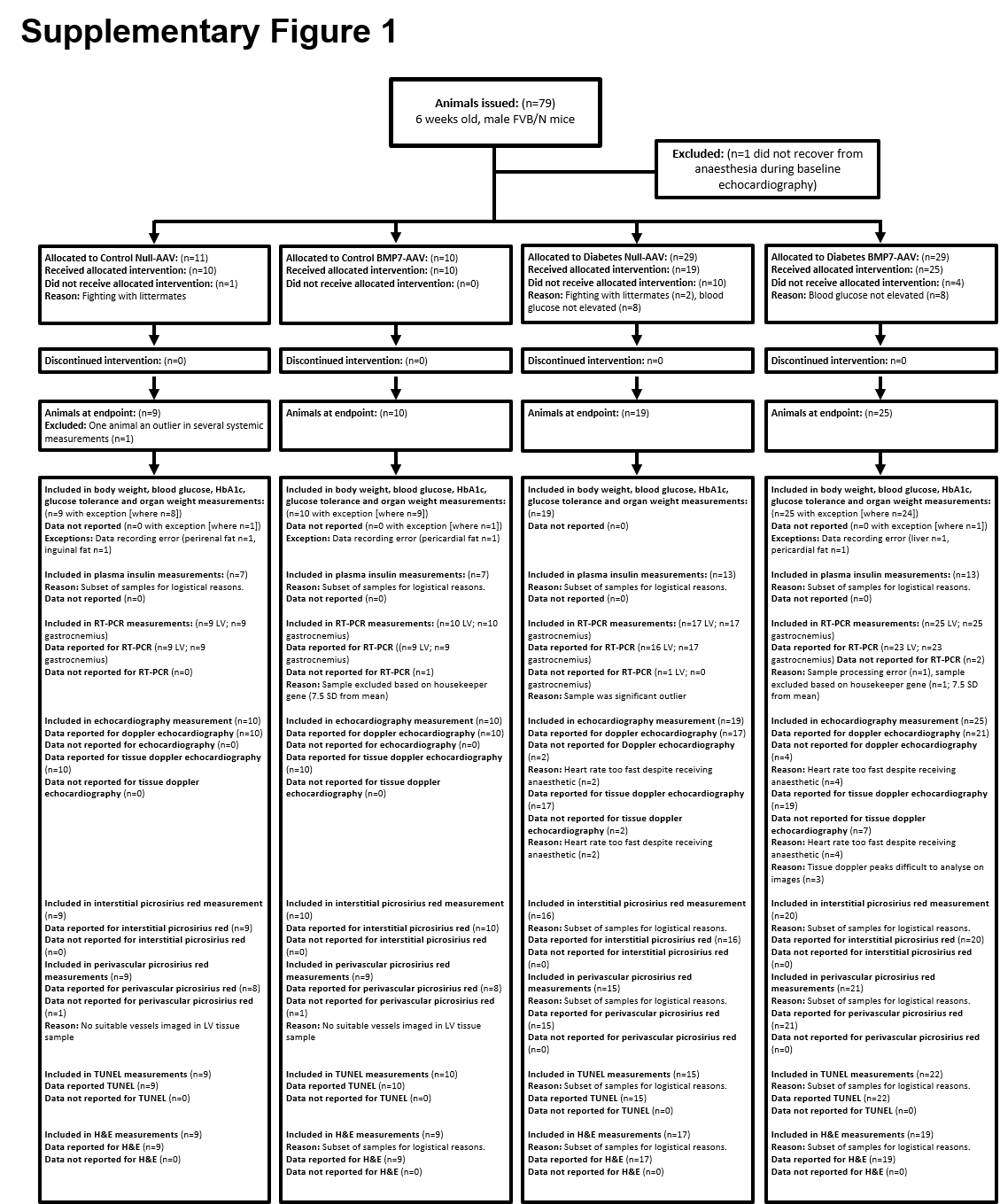

Supplement: Supplementary file 3 [file Image1.TIF]
